# Machine learning approaches in the therapeutic outcome prediction in major depressive disorder: a systematic review

**DOI:** 10.3389/fpsyt.2025.1588963

**Published:** 2025-08-13

**Authors:** Veronica Atemnkeng Ntam, Tatjana Huebner, Michael Steffens, Catharina Scholl

**Affiliations:** Research Division, Federal Institute for Drugs and Medical Devices, Bonn, North Rhine-Westphalia, Germany

**Keywords:** major depressive disorder, machine learning model, outcome prediction, ELSI, decision support system

## Abstract

**Background:**

Various factors impact treatment outcomes in major depressive disorder (MDD), complicating prediction of treatment success. Therefore, applying machine learning (ML) algorithms for therapeutic outcome prediction on the basis of individual patient data has become a promising approach to tailor the treatment strategy in MDD. However, the applicability of such decision support systems in clinical settings has not been sufficiently demonstrated yet. The objective of the evaluation was to assess applicability of currently published ML-approaches for clinical settings in the EU on the basis of quality, ethical, social, and legal criteria.

**Methods:**

We performed a bibliographic search on PubMed and Google Scholar for studies from January 2016 to December 2024 on ML-applications predicting treatment outcomes in MDD. The ML-model applicability was evaluated via information on validation and performance criteria and the compliance with relevant ethical, social, and legal criteria in the EU.

**Results:**

In the 29 publications reviewed, Random Forest (RF) and Support Vector Machine (SVM) were identified as most frequently used ML-methods. Models integrating multiple categories of patient data, demonstrated higher predictive accuracy than single-category models. However, external validation of the applied ML-approaches was limited and due to the early stage of development, compliance with social, ethical and legal standards remains challenging.

**Conclusion:**

A lack of demonstrated generalizability of the evaluated ML-approaches for treatment outcome prediction in MDD and challenges with regulatory compliance in terms of relevant social, ethical and legal aspects do not yet show sufficient applicability and utility for a use in clinical settings in the EU.

## Introduction

According to the World Health Organization (WHO), Major Depressive Disorder (MDD) is a highly widespread condition on a global scale, affecting an estimated number of 280 million individuals (1, 2). The rates of treatment success vary between evidence-based interventions and are influenced by factors such as the patient population, the stage of the disease and e.g. in treatment with several selective serotonin reuptake inhibitors (SSRI) also by pharmacogenetic variants such as of *CYP2D6* and *CYP2C19* (3–6). However, in general a majority of patients do not achieve remission from their depression (7–9). Currently, there is no acknowledged way to anticipate whether a medication will result in a positive response. Thus, present methods for managing MDD primarily depend on trial-and-error sequential treatment tactics (10–12).

Identifying treatment needs in individuals with MDD on the basis of patient indicators early could improve the efficiency of interventions by reducing time spent with unsuitable treatments (13). Personalized approaches including artificial intelligence based models thereby can support tailoring treatments based on each individual’s unique data sets (14). Thus, over the past years, there has been a notable increase in the utilization of machine learning in healthcare (15), including its use for forecasting the results of depression treatments (16, 17). However, a comprehensive framework for quality assessment across an ML-based prediction model to prevent errors in decision making is not yet in place (18).

A clear and transparent development and validation process helps prevent issues like bias and over-fitting, while ensuring the models remain understandable and trustworthy for healthcare professionals (19, 20). However, although the potential of ML in personalized medicine is vast, it also introduces significant challenges that necessitate robust regulation and guidelines (21, 22). In the EU, for the clinical applicability of ML-based decision support systems as diagnostic devices, the quality and safety in terms of an adequate technical and clinical performance for the intended use has to be demonstrated according to the Medical Device Regulation (23). Furthermore, for an application in practice, social, ethical and other legal aspects have to be considered and the clinical utility with respect to MDD outcome prediction needs to be demonstrated (24–26).

To evaluate the clinical applicability and utility of AI approaches in MDD treatment, we conducted a thorough literature analysis of how machine learning is applied to guide therapeutic interventions in MDD. In this systematic review, we highlight the most commonly employed ML-methods and preferred parameters in this context. Additionally, we delineate the performed method validation, provided performance metrics and assess the potential of the ML-models to guide treatment selection on the basis of different categories of patient data. Furthermore, we assess whether compliance with international guidelines with regard to social and ethical considerations and current applicable EU legislation in this context is possible in the use of currently published ML-approaches.

## Methods

### Article search strategy

This systematic review search was conducted using the guidelines and guidance for Preferred Reporting Items for Systematic Reviews and Meta-Analysis: The PRISMA Statements (27). A bibliographical search was carried out using PubMed and Google Scholar. The search terms (antidepressant) AND (prediction response) AND (machine learning models OR machine learning methods) were applied for the publication period of 01^st^ January 2016 to 31^st^ December 2023. The aim was to identify original publications on detailed machine learning approaches for therapeutic outcome prediction in MDD. Thereby interventions such as pharmacological treatment and nonpharmacological treatment were considered. Outcomes considered were remission, response, reduction of symptoms and/or treatment resistant depression. The study designs included clinical trials and randomized controlled trials.

Therefore, further filters applied to PubMed were, “Clinical Trial” and “Randomized Controlled Trial”, excluding all reviews, systematic reviews and meta-analyses. Given the different nature of filters in Google Scholar, only articles without the keywords “Review, “Systematic Review” and “Meta Analysis” were taken into account. Most results from Google Scholar were eliminated as these were conference articles, website articles and posters. Further suitable articles were added through citation screening. In order to update the literature search, a further search with the according search terms and filters was performed in June 2025 to include the year 2024.

### Study selection

The title and abstract from each identified publication were screened, making sure that they addressed MDD as diagnosis, and a machine learning method was used for generating a model on the basis of various patient data or data categories for predicting treatment outcome. All publications, which did not meet the inclusion criteria, were excluded from further evaluations. Studies reporting internal and/or external validation were included. No age or age of onset limit was set in the incidence of the depressive symptoms. Study design was either prospective or retrospective, open-label or controlled without restrictions to allocation blinding or randomization.

### Data extraction

**Table 1** details the profile of information extracted from publications taken into consideration for this review. The identified studies were screened for the ML-method applied, the applied outcome measures and predicted outcome, the medical intervention in MDD, the category of data, validation and technical considerations (class imbalance, missing data approaches, data preparation, feature documentation, source availability/open science applied). Furthermore, performance in treatment outcome prediction was extracted. An overview and definitions of ML-methods and performance metrics are provided in the **Supplementary File 1 Tables 1, 2**.

**Table 1:** Data extraction summary.

Category	Description and/or example
Title	Title of journal
Journal	Publisher
Diagnosis	Major Depressive Disorder (MDD)
Outcome benchmark for depression	Methods used to measure the outcome included the Inventory of Depressive Symptomatology, Self-Report (IDS-SR), Clinical Global Impressions (CGI), Quick Inventory of Depressive Symptomatology (QIDS), Directed Phase Lag Index (DPLI), 17 or 21-item Hamilton Depression Rating Scale (HAMD -17 or-21), Beck Depression Inventory (BDI-II), Montgomer-Asberg Depression Rating Scale MADRS, Quality of life and Function measures, Psychiatric Diagnostic Screening Questionnaire, Physical Health Composite Scores, Perceived Stress Scale (PSS)
Data Category	A data category is a group of related data types organized by analysis goals, like clinical- sociodemographic, molecular biomarker, EEG or MRI data.
Data Source	The origin source of the data used. E.g. study name EMBARC, CAN-BIND.
Sample size	Number of samples included in the testing and training. Can also be represented in percentages.
Machine learning model	Information on machine learning methods used.
Technical details	Evaluation of the availability of source code in the context of open science practices, as well as the adequacy of reporting regarding class imbalance, handling of missing data, data pre-processing procedures, and feature definition.
Performance metric	Information on how the model performance was measured or reported (e.g. AUC ROC (Area under the curve Receiver Operating Characteristics), Sensitivity, Specificity, Accuracy or other).
Validation/Testing	What kind of validation methods were applied (internal, internal-external or external). Use of an independent hold out data set for testing (external validation).
Results	Model performance characteristics.
Full Reference (and Citation)	Supporting unambiguous identification of paper and providing source for citations in tables/figures/text.

### Quality and predictive performance of ML-models in treatment outcome analysis

After screening the selected publications for the type of data used, clinical and sociodemographic data were grouped into a single category for analyses similar to several of the reviewed studies. Furthermore, patient data in the identified publications were categorized in electroencephalography (EEG, including resting state EEG, EEG coherence), magnetic resonance imaging (MRI: structural, resting state, and task-based functional MRI) or molecular biomarker data (genetic, epigenetic, gene expression, metabolites) for this systematic review. Studies applying ML-models on the basis of either only single categories or additionally combined data categories were evaluated separately. Due to the importance of the technical and clinical performance for the applicability of ML-approaches in clinical settings, we focused on the described type of validation and prediction performance in treatment outcome reported in the screened literature.

### Compliance of ML-models with ethical, social and legal aspects to consider for AI algorithms in health care

Relevant ethical and social aspects related to the requirements and necessary restrictions for the safe and beneficial use of AI in health care were identified on the basis of international guidelines such as the general ISO 26000 guidance on social responsibility (28) and the more specific guidance of the World Health Organization (WHO) on “Ethics and governance of artificial intelligence for health” (29). The aim was to evaluate to which extent ethical and social aspects relevant for the use of AI in health care already have been incorporated into EU legislation on the basis of current EU guidelines and which AI applications of our literature search are in accordance with the current regularized criteria.

The ethical key principles in terms of AI use in health specified by the WHO were in accordance with the social issues listed in the ISO 26000 guidance on social responsibility including also the issue of sustainable resource use. For the evaluation whether according principles were met by EU guidelines, criteria were extracted from the specified WHO definitions for each principle (**Supplementary File 2 Table 1**). EU guidelines relevant for the utilization of AI in the health sector such as the General Data Protection Regulation (GDPR) (30), Medical Device Regulation (MDR) (23), and the current version of the European Union Artificial Intelligence (EU AI) act (31), were screened with regard to these overlapping key principles and criteria identified in the ISO and WHO guidance. A comprehensive list of principles and included criteria considered in these regulations is provided in the **Supplementary File 2 Table 2**.

The methods sections of publications identified in the present literature search were screened for applied methodology and assessed in terms of a possible compliance with the above-mentioned criteria relevant for social, ethical and legal considerations (**Supplementary File 2 Table 2**) for AI use in a clinical setting. Published evaluations that did not include external validation were thereby excluded due to insufficient quality.

## Results

**Figure 1:**
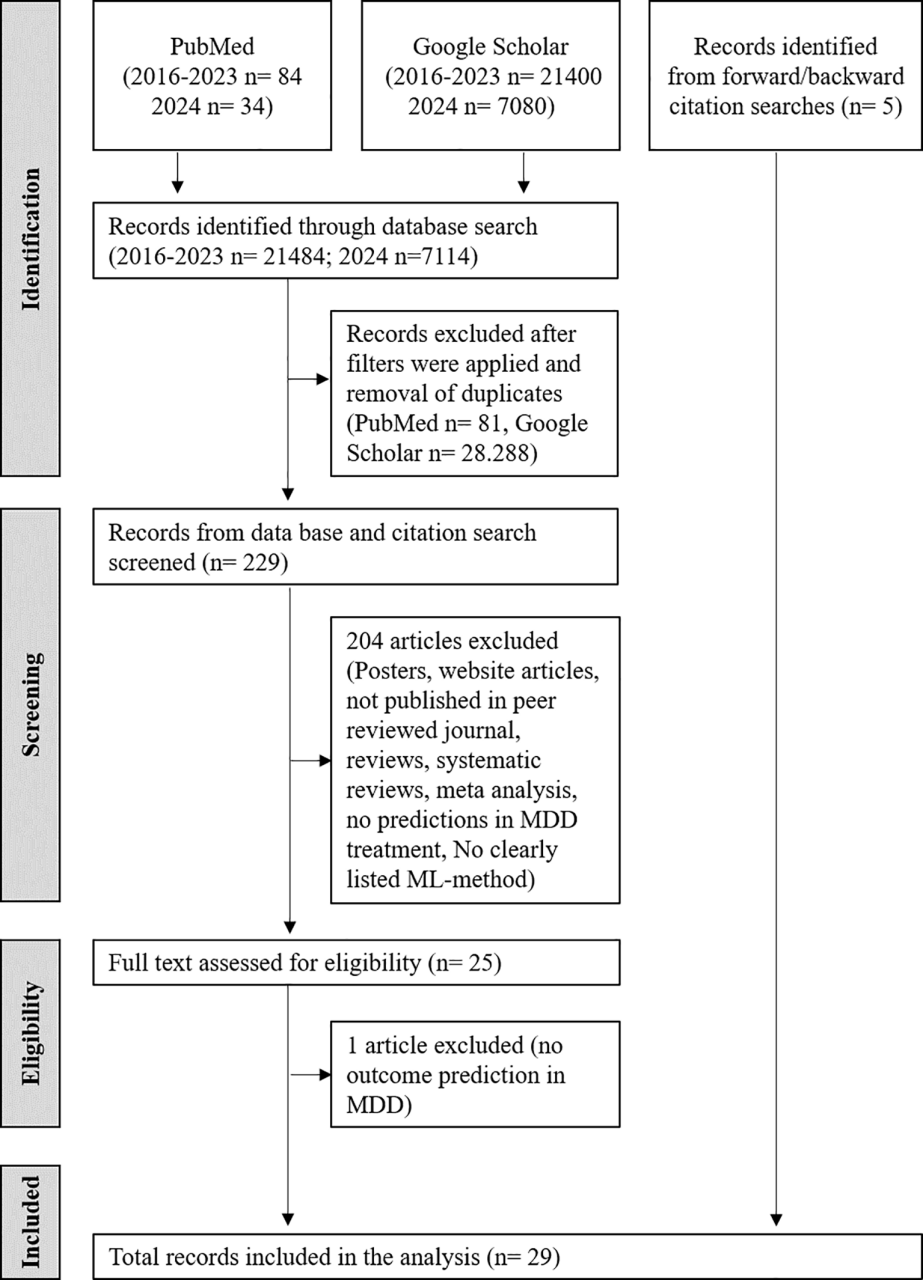
Literature search and selection workflow of eligable records following the guidelines and guidance for Preferred Reporting Items for Systematic Reviews and Meta-Analysis (PRISMA).

To get a broader overview of the ML-approaches that are currently used to predict therapy outcome of patients with MDD, a literature analysis was performed including different types of therapeutic interventions and outcome measures. This literature search yielded a total of 21,484 articles (**Figure 1**, **Supplementary File 2 Table 3**). After consideration of Google Scholar and PubMed filters and removal of duplicates, 34 articles from PubMed and 141 from Google Scholar were considered relevant and selected for further analysis. Screening of titles and abstracts excluded 151 articles due to not meeting the determined criteria or being posters, conference articles, presentations, or web articles, leaving 25 viable articles from PubMed and Google Scholar. The study by Yuelu Liu et al. (2019) (32) appeared to meet the inclusion criteria. However, it was excluded from the review after full text screening because, while it investigated the neural effects of acute dopaminergic enhancement on reward-related brain abnormalities in MDD using machine learning, it did not directly analyze or predict individual clinical responses to antidepressants. In order to update the evaluation, an additional literature search including literature published in 2024 yielded further 7114 publications in June 2025. In the literature search of the year 2024 only 3 articles from PubMed and 51 from Google scholar were screened further. However only one PubMed article provided additional ML-based outcome predictions in MDD for further evaluations (**Supplementary File 2 Table 3**). An additional 5 articles were added through citation screening, resulting in 29 studies used for the systematic review. **Tables 2**, **3** synthesize a diverse array of studies that leverage machine learning techniques to predict outcomes in depression treatment. Treatment outcome was either predicted with respect to TRD (10.3%, N=3) and/or response/change in depressive symptoms (86.2%, N=25) and/or remission/non-remission (20.7%, N=6). However, as measure for the outcome often different benchmarks were used including different clinical scales and methods. The most frequently used psychometric scale for outcome prediction in the reviewed literature was the Hamilton Depression Rating Scale (HAMD) (48.3%, N=14). Overall, 19 different ML-methods were used for approaches in outcome prediction in the screened publications. The most frequently reported methods include Support Vector Machines (SVM, including SVM with radial basis function kernel) in 34.5% (N=10) of studies, and Random Forests (RF) in 55.2% (N=16) of studies. However, in many evaluations several ML-methods were applied and tested.

**Table 2:** Data extracted from identified literature on ML-approaches reported for outcome prediction in pharmacological treatment of MD.

Data Category	Medical Interventions	Data Source	Sample Size	Machine Learning Method	Information on technical details	Validation	External Validation (Validation Cohort)	Depression Symptom Measure	Predicted Outcome	Publication
Clinical (speech analytics)	Psilocybin	Clinical trial enrolees	35 (17 patients, 18 healthy controls)	Gaussian naive bayes	CL-IMB:0, MD:0, DP/FD: 1, SC/OS: 0	7-fold cross-validation	No	QIDS-_16_	Response	Carrillo et al. (2018) (33)
Clinical-sociodemographic data	Citalopram (STAR*D), Escitalopram + placebo Buproprion + Escitalopram Venlafaxine+ Mirtazapine (COMED)	STAR*D, COMED	1941 (STAR*D) 425 (COMED)	EN+gradient boosting machine	CL-IMB:1, MD:1, DP/FD: 1, SC/OS: 0	10-fold cross-validation.	Yes (COMED)	QIDS-SR_16_	Remission	Chekroud et al. (2016) (34)
Clinical-sociodemographic data	Nortriptyline, Escitalopram	GENDEP	793	ENRR	CL-IMB:0, MD:0, DP/FD: 1, SC/OS: 0	10-fold cross-validation, cross-drug analysis	No	MADRS, HAMD-17	Change in depressive symptoms, remission, treatment resistant depression	Iniesta et al. (2016) (35)
Clinical-sociodemographic data	Several medications but not specified	GSRD	552	RF	CL-IMB:0, MD:1, DP/FD: 1, SC/OS: 0	10-fold internal cross-validation	No	MADRS, SDS	Response, treatment resistant depression	Kautzky et al. (2018) (36)
Clinical-sociodemographic data	Citalopram	STAR*D, RIS-INT-93	2782 (STAR*D), 225 (RIS-INT-93)	Penalized logistic regression, RF, GBDT, XGBoost, Elastic Net	CL-IMB:1, MD:1, DP/FD: 1, SC/OS: 0	10-fold cross-validation	Yes (RIS-INT-93)	QIDS-C_16_, QIDS-SR_16_ HAMD-17	Treatment resistant depression	Nie et al. (2018) (37)
Clinical-sociodemographic data	Sertraline, placebo	EMBARC	216	RF, ENRR, BAR	CL-IMB:0, MD:1, DP/FD: 1, SC/OS: 0	10-fold cross-validation	No	HAMD	Change in depressive symptoms	Webb et al. (2019) (38)
Clinical-sociodemographic data	Escitalopram, Citalopram	Combined data from 2 anti-depressant randomized controlled trials (39).	128	RF	CL-IMB:0, MD:0, DP/FD: 1, SC/OS: 0	Monte Carlo simulation for multiple-testing adjustment	No	HAMD, QIDS-SR	Treatment expectancy, change in depressive symptoms	Zilcha-Mano et al. (2019) (40)
Clinical-sociodemographic data	Sertraline, Sertraline+ Mirtazapin, Mirtazapine	SUN☺D	1544	LASSO, ridge, SVM, NN	CL-IMB:0, MD:1, DP/FD: 1, SC/OS: 0	Repeated-fold cross-validation, leave-one-patient-out, leave-one-site-out cross-validation (internal-external validation)	No	PHQ-9	Change in depressive symptoms	Furukawa et al. (2020) (41)
Clinical-sociodemographic data	SSRI, SNRI, Bupropion, and Mirtazapine	Electronic health records from Research Patient Data Registry RPDR	17556	GLM, RF, GBM, DNN	CL-IMB:0, MD:1, DP/FD: 1, SC/OS: 1	Hold-out cross-validation	No	Computer-labeled response effect	Response	Sheu et al. (2023) (46)
Clinical-sociodemographic data	**Psychotherapy (cognitive behavioral** **therapy)** placebo, Fluoxetine, cognitive behavioral therapy + Fluoxetine	TADS	439	Model-based RF	CL-IMB:1, MD:1, DP/FD: 1, SC/OS: 0	Cross-validation	No	CDRS-R	Response	Foster et al. (2019) (42)
Clinical-sociodemographic data	**Psychotherapy (cognitive behavioral** **therapy)** placebo, Fluoxetine, **cognitive behavioral** **therapy** + Fluoxetine	TADS	439	ENR	CL-IMB:0, MD:1, DP/FD: 1, SC/OS: 0	10-fold cross-validation	No	RADS, CDRS-R	Response	Lorenzo-Luaces et al. (2020) (43)
EEG data (resting state EEG)	Escitalopram	CAN-BIND-1	122	SVM	CL-IMB:1, MD:0, DP/FD: 1, SC/OS: 0	Leave-one-site-out cross-validation	No	MADRS	Response	Zhdanov et al. (2020) (44)
EEG data (resting state EEG)	Sertraline, placebo	EMBARC	288	SVM, RF	CL-IMB:1, MD:1, DP/FD: 1, SC/OS: 0	Stratified 5-fold cross-validation, leave-one-site-out cross-validation	No	HAMD-17	Response	Oakley et al. (2023) (45)
EEG data (resting state EEG)	Escitalopram (CAN-BIND-1), Sertraline, placebo (EMBARC)	CAN-BIND-1 EMBARC	125 (CAN-BIND-1) 223 (EMBARC)	K-nearest neighbors	CL-IMB:1, MD:1, DP/FD: 1, SC/OS: 0	Nested k-fold cross-validation	Yes (EMBARC)	MADRS (CAN-BIND-1) HAMD-17 (EMBARC)	Response	Schwartzmann et al. (2023) (46)
MRI data (fMRI and structural MRI)	Sertraline	EMBARC	163	Neural networks	CL-IMB:0, MD:0, DP/FD: 1, SC/OS: 0	Nested k-fold cross-validation	No	HAMD-17	Response	Nguyen et al. (2020) (47)
MRI data (task-based baseline fMRI and structural MRI)	Sertraline	EMBARC	163	Neural networks	CL-IMB:0, MD:0, DP/FD: 1, SC/PA: 0	Nested k-fold cross-validation	No	HAMD-17	Response	Nguyen et al. (2023) (48)
Clinical-sociodemographic data, EEG data	Escitalopram + Bupropion, Escitalopram + placebo, Bupropion + placebo.	Clinical trial enrollees	51	RF, SVM, AdaBoost, MLP, GNB	CL-IMB:1, MD:1, DP/FD: 1, SC/PA: 0	10-fold cross-validation,	No	MADRS	Response	Jaworska et al. (2019) (10)
Clinical data, EEG data (resting state EEG)	Escitalopram, Sertraline, Venlafaxine (extended release)	iSPOT-D	518	GBDT	CL-IMB:0, MD:1, DP/FD: 1, SC/PA: 0	5-fold–stratified cross-validation	No	HAMD-21	Change in depressive symptoms	Rajpurkar et al. (2020) (49)
Clinical-sociodemographic data, MRI data (pretreatment and early-treatment structural MRI)	Sertraline, Placebo	EMBARC	184	RF, PLR	CL-IMB:0, MD:1, DP/FD: 1, SC/PA: 0	5-fold cross-validation	No	HAMD-17	Remission	Bartlett et al. (2018) (50)
Clinical-sociodemographic data, MRI data (pretreatment and/or early-treatment)	Sertraline, Placebo, Placebo +phase2 sertraline	EMBARC	229	XGBoost, SVM, logistic regression	CL-IMB:1, MD:1, DP/FD: 1, SC/OS: 0	Nested cross-validation, randomized K-fold cross- validation, repeated leave-site-out cross-validation (internal-external validation)	No	HAM-D-17	Response, Remission	Poirot et al. (2024) (51)
Clinical-sociodemographic data, molecular biomarker data (genetic, metabolites)	Escitalopram, Citalopram, Bupropion, Venlafaxine, Mirtazapine, combination pharmacotherapies	PGRN-AMPS, CO-MED	264 (PGRN-AMPS) 111 (CO-MED)	PR, XGBoost	CL-IMB:1, MD:1, DP/FD: 1, SC/PA: 1	5-fold cross-validation, cross-trial replication	Yes (CO-MED)	QIDS-C	Response	Joyce et al. (2021) (52)
Clinical-sociodemographic data, molecular biomarker data (epigenetic)	Selective serotonin reuptake inhibitors (SSRIs), non-SSRIs,	Clinical trial enrollees	291	Logistic regression (LR), CART, SVM-RBF, LogitBoost, RF	CL-IMB:0, MD:1, DP/FD: 1, SC/PA: 0	5-fold cross-validation	No	HAMD-17	Response	Chen et al. (2023) (53)
Clinical-sociodemographic data, MRI data, (structural MRI, resting-state and task-based fMRI), molecular biomarker data (genomic, epigenetic, micro RNAs, biochemical markers)	Escitalopram	CAN-BIND-1	192	Penalized regression EN, GBM, RF, SVM, BNA	CL-IMB:1, MD:1, DP/FD: 1, SC/PA: 0	Nested cross-validation	No	MADRS	Response	Sajjadian et al. (2023) (54)

EMBARC, Establishing Moderators and Biosignatures of Antidepressant Response for Clinical Care; STAR*D, Sequenced Treatment Alternatives to Relieve Depression; COMED, Combining Medications to Enhance Depression Outcomes; iSPOT-D, International Study to Predict Optimized Treatment in Depression; SUN☺D, Strategic Use of New Generation Antidepressants for Depression; STEPd, Sequenced Treatment Enhancement Program for Depression; ENAGAGE-2, Engaging self-regulation targets to understand the mechanisms of behavior change and improve mood and weight outcomes in a randomized controlled trial (Phase 2); RAINBOW, Reducing Obesity and Depression Collaborative Care; COPE-D, Comprehensive Observation of Peripheral blood biomarkers and Endocrine Disruptors in Depression; CAN-BIND, Canadian Biomarker Integration Network in Depression; PGRN-AMPS, Pharmacogenomics Research Network - Antidepressant Medication Pharmacogenomics Study; GENEP, Genome-based Therapeutic Drugs for Depression Project; TADS, Treatment for Adolescents with Depression Study; RIS-INT-93, Janssen clinical study RIS-INT-93(Clinical-Trials.govnumberNCT00044681); RPDR, Research Patient Data Registry; MGB, Mass General Brigham Healthcare System; NIMH, National Institute of Mental Health. These abbreviations represent various clinical trials, research projects, and data registries focused on understanding and treating depression. The machine learning methods listed in the table are Least Absolute Shrinkage and Selection Operator regression (LASSO), Support Vector Machine (SVM), Random Forest (RF), Gradient Boosting Machine (GBM), Logistic Regression with Elastic Net Regularization (Logistic EN), Penalized Logistic Regression (PLR), Neural Networks (NN), Ridge Regression (RR), General Linear Models (GLM), Gaussian Process (GP), Extreme Gradient Boosting (XGBoost), k-Nearest Neighbours (k-NN), Bayesian Network Analysis (BNA), Decision Trees (DT), Monte Carlo Simulation (MCS), Cluster Analysis (CA), Elastic Net Regularized Regression (ENRR or Elastic Net or EN), Quantitative Receiver Operating Characteristic algorithm (QROC), Bootstrapped Regression (BR). Selective Serotonin Reuptake Inhibitors (SSRIs), Serotonin-Norepinephrine Reuptake Inhibitors (SNRIs). Inventory of Depressive Symptomatology, Self-Report (IDS-SR), Clinical Global Impressions (CGI), Hamilton Depression Rating Scale (HAMD-17), Quick Inventory of Depressive Symptomatology (QIDS), Directed Phase Lag Index (DPLI), Structured Clinical Interview for DSM-IV-TR Axis I Disorders (SCID), 16-item self-report QIDS (QIDS-SR), Beck Depression Inventory (BDI-II), Frequency, Intensity, and Burden of Side Effects Rating (FIBSER), Quality of Life and Function Measures, Montgomery-Åsberg Depression Rating Scale (MADRS), Mood and Anxiety Symptom Questionnaire (MASQ), Clinician-Rated Quick Inventory of Depressive Symptomatology (QIDS-C), Perceived Stress Scale (PSS), Hamilton Rating Scale for Depression (HRSD), Hamilton Anxiety Rating Scale (HARS), Patient Health Questionnaire (PHQ-9), Children’s Depression Rating Scale-Revised (CDRS-R), Clinical Global Impressions of Severity (CGI-S). Information on technical details (0 = not addressed, 1 = addressed): CL-IMB, Class imbalance; MS, Missing data; DP/FD, Data preparation and feature documentation; SC/OS, Source code availability/open science.

**Table 3:** Data extracted from identified literature on ML-approaches reported for outcome prediction in non-pharmacological treatment of MDD.

Data Category	Medical Interventions	Data Source	Sample Size	Machine Learning Method	Information on technical details	Validation	External Validation(Validation Cohort)	Depression symptom measure	Predicted Outcome	Publication
Clinical-sociodemographic data	Psychotherapy (cognitive behavioral therapy, interpersonal psychotherapy)	Clinical trial enrolees (RCT)	182	Regression model	CL-IMB:0, MD:1, DP/FD: 1, SC/OS: 0	5-fold cross- validation	No	BDI-II	Change in depressive symptoms	Bronswijk et al. (2021) (55)
Clinical-sociodemographic data	Psychotherapy (cognitive behavioral therapy, interpersonal psychotherapy)	STEPd, FreqMech	151 (STEPd) 200 (FreqMech)	RF, regression model	CL-IMB:0, MD:1, DP/FD: 1, SC/OS: 0	5-fold-cross- validation,	Yes (cross-trial-validation: STEPd, FreqMech)	BDI-II	Change in depressive symptoms	Bronswijk et al. (2021) (56)
Clinical-sociodemographic data	Psychotherapy (digital intervention: online supportive expressive therapy with feedback from a therapist, information related to stress management and coping)	Study (57)	632	NN, RR, RF,GLM, GP, XGBoost, KNN, SVM	CL-IMB:0, MD:0, DP/FD: 1, SC/OS: 0	Out-of-sample nested cross-validation	No	PHQ-9	Changes in depressive symptoms	Jacobson et al. (2021) (58)
Clinical-sociodemographic data	Psychotherapy (problem-solving therapy, supportive therapy)	COPE-D	221	RF	CL-IMB:1, MD:1, DP/FD: 1, SC/OS: 0	Sensitivity analysis	No	HAMD	Response/ remission	Solomonov et al. (2021) (59)
Clinical-sociodemographic data	Psychotherapy (problem solving therapy)	ENAGAGE-2, RAINBOW	60 (ENAGAGE-2) 175 (RAINBOW)	SVM, RF, AdaBoost, GBT, LR	CL-IMB:0, MD:1, DP/FD: 1, SC/OS: 0	5-fold cross- validation	Yes (RAINBOW)	PHQ-9	Remission	Kannampallil et al. (2022) (60)
EEG data (Resting state EEG, EEG coherence)	Transcranial magnetic stimulation	Clinical trial enrolees (prospective unblinded trial)	35	LASSO, SVM	CL-IMB:0, MD:1, DP/FD: 1, SC/OS: 0	Leave-one-site-out cross-validation	No	IDS-SR	Response	Zandvakili et al. (2019) (61)

FreqMech, Frequency and Change Mechanisms of Psychotherapy for Depression; STEPd, Sequenced Treatment Enhancement Program for Depression; ENAGAGE-2, Engaging self-regulation targets to understand the mechanisms of behavior change and improve mood and weight outcomes in a randomized controlled trial (Phase 2); RAINBOW, Reducing Obesity and Depression Collaborative Care; COPE-D, Comprehensive Observation of Peripheral blood biomarkers and Endocrine Disruptors in Depression; LASSO, Least Absolute Shrinkage and Selection Operator; SVM, Support Vector Machine; RF, Random Forest; NN, Neural Network; RR, Ridge Regression; GLM, Generalized Linear Model; GP, Gaussian Process; XGBoost, Extreme Gradient Boosting; KNN, K-Nearest Neighbours; AdaBoost, Adaptive Boosting; GBT, Gradient Boosted Trees; LR, Logistic Regression. IDS-SR, Inventory of Depressive Symptomatology - Self-Report; BDI-II, Beck Depression Inventory-II; PHQ-9, Patient Health Questionnaire-9; HAMD, Hamilton Depression Rating Scale. Information on technical details (0 = not addressed; 1 = addressed), CL-IMB, Class imbalance; MS, Missing data; DP/FD; Data preparation and feature documentation; SC/OS, Source code availability/open science.

Medical interventions primarily involve pharmacotherapy with antidepressants in 72.4% (N=21) of the screened literature. Thereby, selective serotonin reuptake inhibitors such as citalopram, escitalopram, fluoxetine, and/or sertraline are represented in all of these publications in mono- or combination pharmacotherapy (65.5%, N=19) or in combination with psychotherapy (6.9% N=2). Further pharmacotherapy-based interventions identified were norepinephrine–dopamine reuptake inhibitors such as bupropion in mono- and combination pharmacotherapy 10.3% (N=3), serotonin and norepinephrine reuptake inhibitors represented by venlafaxine) in mono- and combination pharmacotherapy (10.3% N=3), tricyclic (nortriptyline, N=1) and tetracyclic antidepressants (mirtazapine in mono- and combination pharmacotherapy (13.8%, N=4)). Furthermore, in one publication the psychedelic compound psilocybin which is not an approved drug in the EU, was used for pharmacological therapy in MDD (**Table 2**). Non-pharmacological interventions only (**Table 3**) were mainly represented by different psychotherapy approaches identified in 17.2% (N=5) of the screened publications. Transcranial magnetic stimulation was reported as non-pharmacological intervention in only one publication.

Internal validation techniques enhance the reliability of the model performance evaluation (62). The validation techniques applied in the screened studies were predominantly described as rigorous, with cross-validation methods such as predominantly k-fold cross-validation (62.1%, N=18), nested cross-validation (20.7%, N=6), and leave-one-out cross-validation (17.2%, N=5). However, external validation, to ensure the generalizability of the models, is reported in 6 studies only.

Most studies explicitly addressed missing data, commonly through exclusion or imputation. While implementation details are sometimes limited, the problem is clearly recognized across studies. Fewer studies discussed class imbalance. Very few by oversampling, most of them stratified cross-validation (**Tables 2**, **3**), but only rarely was the issue addressed in-depth or through appropriate metrics like Area Under the Precision-Recall Curve (AUC-PR). This highlights a methodological gap. Across all studies, the description of data preprocessing steps and the features included in the models was consistently clear and well-documented. However, very few studies provided public access to code or data. Still, most described models and variables in sufficient detail to enable partial reproducibility. Although several studies described their methodology to prevent bias to some extent, they did not discuss potential biases in-depth. Ensuring transparency, explainability and intelligibility however is a key principle to be considered for implementation in clinical settings (29, 63).

### Outcome prediction on the basis of a single data category

**Figure 2:**
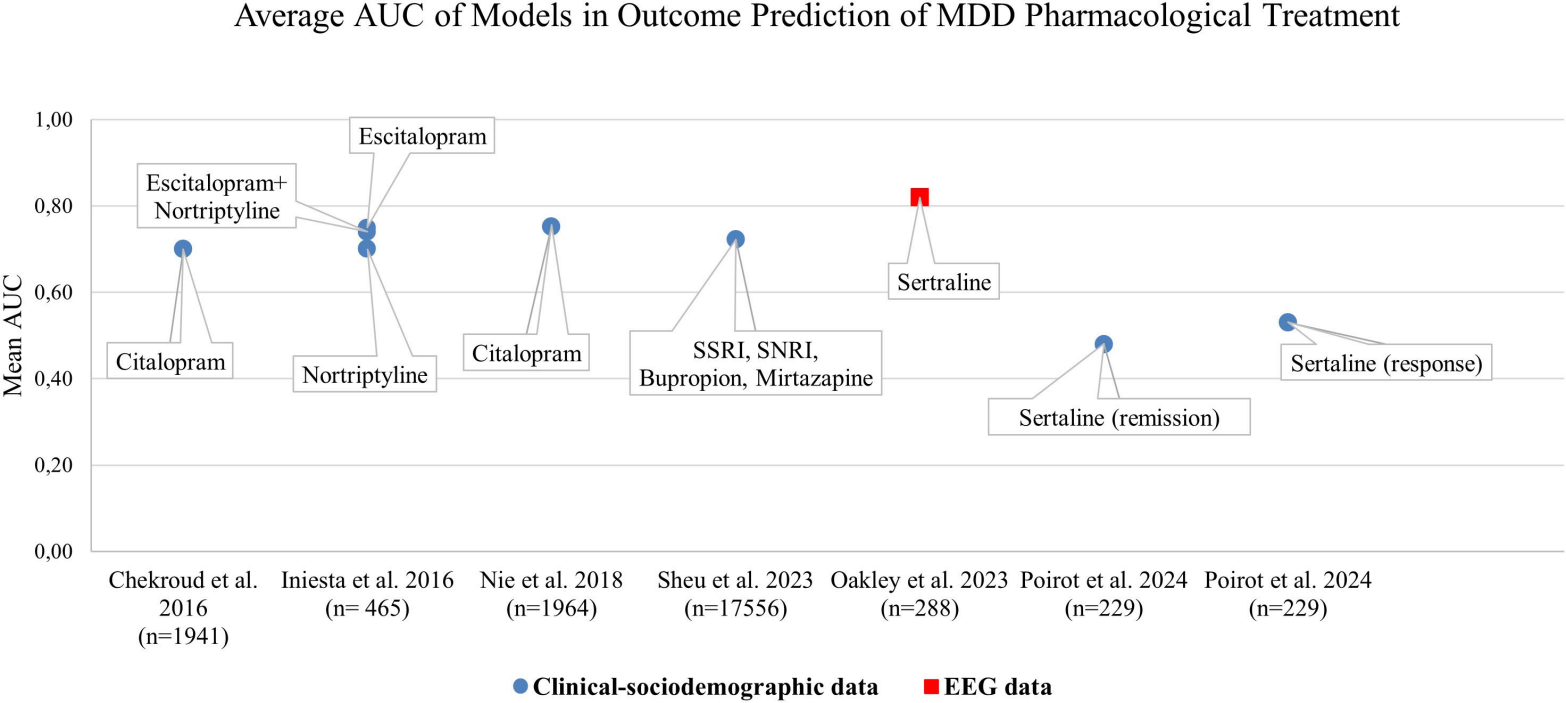
Mean Area Under the Curve (AUC) values across ML-methods internally validated on pharmacological treatment groups of various studies from 2016 to 2024.The diagram highlights the models applying a single patient data category. Chekroud et al. (2016): prediction of remission; feature selection applied. Iniesta et al. (2016): prediction of remission; largest predictor set with random patient allocation. Nie et al. (2018): prediction of treatment resistant depression, full set of features considered. Sheu et al., 2023: prediction of response; likelihood score and inclusion of deep-learning imputed labels not considered. Oakley et al. (2023): prediction of response, feature selection applied. Poirot et al. (2024) prediction of pre-treatment response, pre-treatment remission; largest predictor set without selection.

A majority of identified literature on ML-approaches for treatment outcome predictions was based on a single data category as defined for this review such as EEG or MRI or Clinical-sociodemographic data. Thereby, primarily the data category clinical-sociodemographic was represented. Overall, 34.5% (N=10) of identified publications reported approaches based on clinical-sociodemographic data in outcome prediction of pharmacotherapy or pharmacotherapy and psychotherapy (**Table 2**) and 17.24% (N=5) in psychotherapy only (**Table 3**). **Figure 2** juxtaposes the performance metric AUC reported in publications on prediction models internally validated in terms of pharmacological treatment of MDD applying one data category only. Predominantly, AUC was reported in evaluations of the single data category clinical-sociodemographic in antidepressant treatment. In general, reported average AUC for identified single data category prediction models applied in antidepressant treatment was about ≥0.7. However, the approach by Poirot et al. (2024) shows a clearly lower performance for response and remission (**Figure 2**, **Supplementary File 3 Table 1**). In non-pharmacological treatment the AUC values were only reported by Zandvakili et al. (2019) for outcome prediction in Transcranial magnetic stimulation (AUC (Alpha coherence): 0.83; AUC (Theta coherence): 0.69) and by Kannampallil et al. (2022) for outcome predictions in psychotherapy (AUC (baseline clinical and patient-reported data): 0.719; AUC (baseline and data after 2 months of problem solving therapy): 0.744. Furthermore, performance metrics such as accuracy, sensitivity and specificity were often reported (**Supplementary File 3 Table 1**) (44, 46, 51, 59). F1 or R^2^ were predominantly applied to report performance in models using the data category EEG or MRI, and therefore were identified less frequently (43, 45, 47, 48). Model performance based on different methods was often higher with a larger set of selected patient variables (35, 36). However, Nie et al. (2018) showed that dependant on the method applied, the increase of performance with additional variables reached a plateau with a differing number of top variables. Also Iniesta et al. (2016) showed that additional variables can improve performance of outcome prediction only to a certain point. However, this impact was dependent on predicted outcome, applied pharmacological therapy and variable combination. Furthermore, in several publications on the basis of one data category, the combination of baseline data and data in the course of treatment increased performance such as AUC and/or accuracy (44, 51, 60).

### Effect of combining patient data categories in ML-approaches

Utilizing diverse patient data enables ML-models to capture complex patterns, leading to improved predictive accuracy and a more personalized approach to treatment for individuals with MDD (34, 64). Thereby, adding extra data such as combining different clinical and demographic patient variables (35) or combining measurements at baseline and in the course of treatment in general improved performance metrics such as AUC of predictive models for e.g. depression remission in previous studies (44, 51, 60). In several of the evaluated publications (**Table 2**), also ML-models that analyzed more than just one category of patient data were described (10, 49–54).

Joyce et al. (2021) assessed whether ML-approaches trained on data of patients with antidepressant monotherapy can improve outcome predictions in combination with antidepressant pharmacotherapy when additional multi-omics measures are incorporated in comparison to clinical-sociodemographic and metabolomic data (molecular biomarker data) only (52). The accuracy achieved in external validation of the metabolomics model was 75.3% (p = 0.026) for both penalized regression and XGBoost. When utilizing the multi-omics model, the accuracy decreased to 73.2% (p = 0.085) applying XGBoost and increased to 77.5% (p < 0.01) with penalized regression. For AUC similar tendencies were observed (**Supplementary File 3 Table 2**).

Thus, the extended multi-omics model slightly decreased or only slightly increased prediction performance in terms of accuracy and AUC when applied on patients with combination antidepressant pharmacotherapies. The additional molecular biomarker data (6 functionally validated SNPs), therefore did not show a high impact in the cross-trial replication experiment by Joyce et al. (2021) and also in internal validation the impact on performance was low.

However, the additional incorporation of different types of data categories and feature selection showed a higher impact on the improvement of predictive performance in the analyses by Jaworska et al. (2019) (10) and by Poirot et al. (2024) (51). Jaworska et al. (2019) showed that Model 3 which combined EEG and clinical-demographic data categories with selected most predictive features for week 12 treatment response/non-response performed better than models on the basis of single data categories with predictive feature selection (**Supplementary File 3 Table 3**). The model applying the most predictive variables from feature selection of each category achieved high sensitivity and specificity and overall accuracy of 88%. Thereby, the approach using Random Forest (RF) was superior in terms of quality metrics across all the machine learning methods.

Poirot et al. (2024) demonstrated that multimodal approaches in remission and response prediction with pre-treatment MRI and clinical data predominantly outperformed unimodal models. However, when early treatment data was incorporated the effect was not observed for unimodal models applying clinical-sociodemographic data only to predict remission on the basis of low scientific evidence predictors or response with a large predictor set without selection in response prediction. In general, the use of a selected set of high scientific evidence predictors in multimodal approaches showed a higher performance. Also, Chen et al. (2023) (53) showed that combining different data categories tends to improve prediction performance. RF performed overall best across the different models using clinical-sociodemographic and/or molecular biomarker data (methylation levels of 38 methylated sites of tryptophan hydroxylase 2 genes via SNPs). Specifically, it achieved the highest AUC and accuracy when combining the molecular biomarker and clinical-sociodemographic data (**Supplementary File 3 Table 4**, Model 3). Furthermore, feature selection applying Recursive Feature Elimination (RFE) with random forest in general improved performance in terms of accuracy in the single and the multiple category approach. However, significance was obtained only in the molecular biomarker model and the combination model.

Sajjandian et al. (2023) (54) performed single and multiple data category analyses on clinical- sociodemographic data, MRI (multimodal and functional) and molecular biomarker data with and without feature selection. Thereby, a set of 134 baseline variables preselected on the basis of relevant published evidence of predictive value and a large set of 1152 baseline variables without the requirement of according evidence were used. For single and multiple data category analyses, the approaches including the smaller evidence-based set of variables in general showed better mean balanced accuracy than the according approaches with the large variable set. However, in the large variable set without prior evidence-based preselection, correlation-adjusted t-score feature selection led to a modest improvement of mean balanced accuracy. The inclusion of 80 variables representing measurements at week 2 of treatment in addition to the evidence based 134 variables mainly improved mean accuracy in single and multiple data category analyses (**Supplementary File 4 Table 1**). Furthermore, an increase of mean accuracy was observed with the increase of combined data categories over all variable set approaches.

In the application of baseline variables only (134 preselected baseline variable set, feature selection), the best-performing ML-method in terms of accuracy was SVM in Model 4 combining all data categories and in Model 2 applying molecular biomarker data (**Supplementary File 4 Table 1**). For the combined data of baseline (134 preselected, evidence-based baseline variable set, feature selection) and week 2 measurements (80 variables) in the course of treatment, the most accurate predictive models were a Naive Bayes model (CAT score) that utilized clinical data, an Elastic Net model (Embedded + CAT score) applying all data categories and a random forest model (Embedded + CAT score) that incorporated clinical data.

In sum, the combination of additional data categories improved accuracy with a higher impact than the addition of variables of the same data category in the identified literature for this review (35, 51, 52, 54).

### Compliance of published AI applications with current social, ethical and legal aspects

A majority of extracted and screened criteria representing WHO ethical key principles (**Supplementary File 2 Table 1**) agreeing with ISO issues on social responsibility are addressed by current EU regulations that are translated into national legislation in the EU (**Supplementary File 2 Table 2**). Results on external validation applying new independent data sets of different studies were provided by only six of the identified publications. However, only five showed successful validation (**Table 4**). Though, the evaluated five publications provide performance data on external validation, which is a necessary step toward an application in clinical settings, they still describe ML-models in the development stage. Therefore, several of the relevant principles that cover ethical and social aspects that need to be considered for a potential medical product according to EU legislation could not be assessed yet.

**Table 4:** Compliance of externally validated ML-models with current social, ethical and legal aspects applicable in the EU.

Criteria (social, ethical and legal aspects)	Chekroud et al. (2016) (34)	Nie et al. (2018) (37)	Joyce et al. (2021) (52)	Kannampallil et al. (2022) (60)	Schwarzmann et al. (65)
Protecting human autonomy					
1. Human control	NA	NA	NA	NA	NA
2. Data protection	NA	NA	NA	NA	NA
3. Accordance to the human rights	NA	NA	NA	NA	NA
4. Informed and valid consent ensured	✔	✔	✔	✔)	✔
Promoting human well-being and safety and the public interest					
1. Human well-being	(✔)	(✔)	(✔)	(✔)	(✔)
2. Human safety	**Citalopram +Placebo** Acc.: 59.6%, Sensitivity: 49.4%, Specificity: 70.8% **Escitalopram+Buproprion** Acc.:59.7%, Sensitivity: 56.1%, Specificity: 63.2% **Venlafaxine+Mirtazapine** Acc.:51.4% Sensitivity::38.9, Specificity: 64.7	**Citalopram** AUC: 0.60-0.73 Acc.: 82-86%, Sensitivity: 88-92%, Specificity: 36-44%	**Venlafaxine +Mirtazapine/Escitalopram +Bupropion** **Metabolomics** AUC: 0.75-0.84 Acc.: 75.3% Sensitivity: 65-73% Specificity: 79-93% **Multi-omics** AUC: 0.74-0.86 Acc.:73.2-77.5% Sensitivity:71-80% Specificity: 62-88%	**Psychotherapy** **Baseline** (clinical and patient-reported data): AUC: 0.670 Acc.:58.3% Sensitivity:71% Specificity:55.6% **Baseline+2 month model** AUC: 0.709 Acc.: 62.3%, Sensitivity: 71.0% Specificity: 60.4%	**Sertraline** Acc.: 63.7%, Sensitivity: 58.8%, Specificity: 68.5% **Placebo:** Acc.: 48.7% Sensitivity: 50.0% Specificity: 47.3%:
3. Public interest:	(✔)	(✔)	(✔)	(✔)	(✔)
Ensuring transparency, explainability and intelligibility					
1. Intelligibility/explainability:	✔	✔	✔	✔	✔
2. Transparency	✔	✔	✔	✔	✔
Foster responsibility and accountability					
1. Responsibility	NA	NA	NA	NA	NA
2. Accountability	NA	NA	NA	NA	NA
Ensure inclusiveness and equity					
1. Equity	NA	NA	NA	NA	NA
2. Inclusiveness	NA	NA	NA	NA	NA
3. Diversity	(✔)	NA	✔	✔	NA
4. Protection from stigmatization or discrimination	NA	NA	NA	NA	NA
Promote artificial intelligence that is responsive and sustainable					
1. Responsive AI	NA	NA	NA	NA	NA
2. Sustainability	NA	NA	NA	NA	NA
3. Efficiency	NA	NA	NA	NA	NA

NA, not available; Acc., Accuracy; (✔), accordance can be assumed.

Although all ML-models developed and described for the prediction of either treatment response and/or TRD (37, 46), non-remission or remission (34, 60) prior to treatment initiation or in the early phase of treatment (60) in MDD aim to contribute to human well-being, their capability to provide a real health benefit and to be of public interest needs to be demonstrated. In four publications it was mentioned that an informed consent for the use of the data was available (37, 46) or they referred to information on trial registration or previous publications of study protocols that confirmed an available written informed consent (60) while one publication (34) mentioned data use via a limited access data use certificate. Still, whether a consent policy will be applied with the use of the ML-applications could not be deduced. Only Joyce et al. (2021) and Kannampallil et al. (2022) indicate the inclusion of several ethnic groups in the training data set, however the data sample was small. Chekroud et al. (2016) indicate “White” and “Black or African American” as applied variables. However, the aspect diversity was not addressed comprehensively. All publications showed that the applied ML-models were methods of supervised learning and were concordant with the principles “Intelligibility/explainability” and “Transparency”. Furthermore, data sources, the process of obtaining the data, the method of data processing, data inclusion and exclusion and a discussion of the data bias and limitations of the ML-models were addressed. The aspect “Human safety” was addressed by an external validation and performance assessment of the ML-model in MDD. Performance was reported in all evaluated publications (34, 37, 46, 52, 60) at least in terms of accuracy, sensitivity and specificity in the prediction of the according models. In most published ML-models the evaluated metrics showed above chance performance for prediction accuracy. However, the external validation by Chekroud et al. (2016) could not show an above chance performance in a Venlafaxine plus Mirtazapine treatment cohort in terms of accuracy. Furthermore, all models externally validated by Nie et al. (2018) could not reach specificity higher than 44% in the prediction of citalopram treatment resistant depression though accuracy and sensitivity were high. Only Chekroud et al. (2016) compared model prediction with treatment outcome prediction by clinicians. Here, prediction performance by 23 clinicians for 26 STAR*D patients was below chance and the ML-model showed a better performance.

## Discussion

The heterogeneity and complexity of MDD aggravate the estimation of the course of treatment and thus result in the requirement to include a variety of different patient data for its assessment (66). For such multifaceted analyses, however clinicians will need tools to integrate the varying patient information for guiding treatment decisions. In the EU, algorithms that are used as decision support systems in clinical settings have to be validated like diagnostic devices according to the Medical Device Regulation (MDR) (23). Furthermore, the General Data Protection Regulation (30) and, from August 2026, the new EU AI Act (31) also apply when a use on patient data in patient care is executed. Therefore, several aspects in terms of performance quality, data safety, transparency and further social and ethical considerations must be taken into account when using machine learning to guide treatment for MDD (28, 29). In this systematic review we evaluated how clinical applicability in terms of technical and clinical performance, accordance with social, ethical and legal issues relevant in the EU and utility of AI models for the therapeutic outcome prediction in MDD have been addressed and demonstrated so far in published literature.

We could show that all evaluated publications addressed the issue of technical performance by applying some sort of internal validation and, in a majority, also reporting performance metrics such as accuracy and/or AUC, sensitivity, specificity and in some cases also other quality metrics such as R² or the F1 score. The findings from the performance metric evaluation of various ML-methods across different data categories indicate that the incorporation of multiple categories of patient data mainly leads to enhanced model performance. Thus, across the reviewed studies, the inclusion of a broader set of data categories—such as clinical-sociodemographic, EEG, MRI and molecular biomarker data—demonstrated the ability of ML-models to improve the accuracy of predictions related to treatment outcomes for Major Depressive Disorder (MDD) (10, 35, 51–54, 60). However, some approaches were more successful particularly when escitalopram or citalopram treatment was involved, indicating that the benefit of adding data may vary dependent on the treatment being predicted (10, 34, 35). Another systematic literature evaluation by Lee et al. in 2018 supports our findings showing that predictive models that integrate multiple categories of data performed better than models applying single data categories of adults with depression. Furthermore, also Lee et al. (2018) reported issues on heterogeneity consistent to our observations especially in terms of validation approaches, applied data and feature selection (67). In terms of other mental disorders Pigoni et al. (2025) showed that overall accuracy of predictive models is increased with the use of more than one data category (68). A previous scoping review by Kline et al. (2022) on ML in precision health showed that multimodal approaches and unimodal approaches were compared only in few publications. However, in cases where a comparison was provided predictive accuracy was increased with the multimodal approach (69). The incorporation of multiple categories of patient data is also supported by studies focusing on other health conditions such as neurodegenerative diseases or cancer. (70–72). Furthermore, similar to our observations on the basis of the reviewed ML-approaches, the use of feature selection techniques often further optimized the model performance also in predictions focusing on e.g. cancer therapy (73–75).

Although evidence of the importance of genetic or pharmacogenetic information in MDD treatment outcome has increased (76–78), only 10.3% (N=3) of the reviewed publications have incorporated such molecular biomarker data in their prediction models in MDD. However, several of the published ML-model evaluations showed a better performance or, dependant on the applied method, a trend of improved performance metrics when biological biomarkers such as genetic characteristics were considered (52, 53). However, none of the reviewed studies included data on relevant pharmacogenetic variants of e.g. *CYP2D6* or *CYP2C19* in ML-models for outcome prediction in SSRI treatment, although in previous studies sufficient evidence of a potential impact on treatment with several SSRI has been demonstrated to provide genotype based treatment and dosing recommendations (79). While adding and combining various data categories may help to improve prediction performance, Sajjadian et al., 2023 (54) showed that adding a large number of data without preselection on the basis of scientific evidence in terms of relevance for prediction and without feature selection does not necessarily lead to better accuracy. Also, in some approaches with preselection and/or feature selection applying one data category only such as clinical-sociodemographic data showed similar or even higher performance than approaches of combined models (51, 54).

According to the MDR, apart from analytical or technical performance also clinical performance should be assessed and should be suitable for the intended use of ML-models in MDD to ensure a safe and efficacious application by clinicians for therapeutic outcome prediction. However, only five publications provided information on a successful external validation to demonstrate robustness relevant for clinical performance (34, 37, 46, 52, 60). Thereby, only two publications reported external validations with high accuracies of >75%, moderate to high sensitivity of >70%, and only one in parallel a high specificity of 88% in outcome prediction for an independent data set (**Table 4**). However, sample size of the training data set in these cases was low. Appropriate performance values for MDD were not discussed in any of these publications and an above chance performance was still presented as successful (34). This review highlights that external validation as a crucial phase in the qualification process for a clinical application is underrepresented in current literature.

For clinical applicability, it should be furthermore demonstrated that the AI application can be utilized according to current legislation also taking into account social and ethical aspects to ensure public acceptance (63, 80). We identified and presented social and ethical aspects that need to be considered according to current EU legislation when AI models are used as diagnostic devices to assist MDD treatment outcome prediction as clinical decision support systems. However, as the ML-applications presented in current literature are still in the stage of development, many of these aspects cannot apply. Still, important aspects such as safety in terms of reporting performance metrics and to some extent also intelligibility, explainability, and transparency were met by all publications that presented results on successful external validation. This shows a potential for a future application in healthcare.

The demonstrated heterogeneity across the published studies in terms of study design, sample size, validation approaches, applied ML methods, number and type of applied data categories (**Tables 2**, **3**) and the approaches of feature selection aggravate generalizability (81). This also provides a barrier to clinical implementation of such ML-methods as regulators in the EU require evidence of consistent performance across settings and populations in the course of post-market surveillance. Furthermore, for implementation of such decision support systems in clinical settings and to maintain real-world clinical performance, updating of ML-models in order to enable them to operate according to the intended use, a longitudinal validation and thereby consistent demonstration of compliance with current legislation is necessary (31).

Currently, there are no standards for predictive performance of ML-models in the therapeutic outcome prediction in major depressive disorder. Such standards could provide guidance to manufacturers in the development process of decision support systems and to regulators in terms of decision-making according to current legislation. However, due to the high burden for MDD affected individuals, their social environment and on health care (82), a thorough and careful evaluation by medical experts taking into account the intended use of the decision support system is necessary prior to determining appropriate metrics and levels of predictive performance (18, 83),. Thereby, the consequences of false positive and false negative results in the context of use and of specific predicted outcomes should be considered (84). However, also the consequences of not using the application should be taken into account, if evidence is provided in real-world practice that the decision support system demonstrates a clinical benefit. Thereby, comparative evaluations showing whether externally validated ML-based approaches surpass current approaches by healthcare providers could deliver such evidence. This could support implementing thresholds that would constitute acceptable levels of predictive performance. However, a demonstration that the application of an ML-model in real-world settings is useful in MDD outcome prediction was not sufficiently provided by any of the publications reporting at least an external validation of the according ML-models. Only Chekroud et al. (2016) compared AI-aided outcome prediction with AI-unaided prediction by 23 clinicians on a small sample of patients. Therefore, clinical utility has not been sufficiently addressed by an appropriate performance comparison with currently applied methods in real-world settings to show a value and benefit for patient care. It needs to be assessed which prediction performance of an AI-based decision support system compared to a prediction by clinicians can be regarded as useful. Thereby, also, the expert level of clinicians should be taken into account and that the aspect of human control can be met in the application of the decision support system despite of the complexity of underlying patient data. However, most of the published ML-models providing an above chance prediction performance in this systematic review are currently not suitable for a use in clinical settings in the EU as they do not meet all requirements in terms of clinical applicability and clinical utility. Furthermore, successful implementation of ML-models as decision support systems can also depend on stakeholder acceptability (85). An integration of patients’ and clinicians’ perspectives enables an important insight into the end-user environment and helps to identify needs, challenges and barriers for application according to the intended use and for adoption in real-world settings. Therefore, it is crucial to consider stakeholder feedback in the design and development process (86). Usability and whether the application can be applied in accordance with ethical, social and legal aspects such as explainability and transparency can be tested involving patients and clinicians in prototyping cycles (86–88). Furthermore, interviews or surveys could be integrated in the development, validation process or in pilot testing to evaluate user satisfaction and perceived utility (89, 90). However, none of the evaluated studies addressed the patients’ and clinicians’ perspectives in terms of the application of such tools for MDD therapy management in clinical care.

For this literature evaluation, the search string was chosen to screen a large number of publications on ML-based outcome predictions in MDD. Still, the search terms used may not represent all current literature in terms of the objective of this systematic review. Limitations of the literature search were that two data bases including Pubmed and Google Schoolar were applied for the search while data bases such as e.g. EMBASE, Medline, Scopus or PsycINFO were not applied. To increase the quality of the assessment, only original publications of clinical trial evaluations were included. However, few publications met the required criteria and provided detailed descriptions of ML-approaches applied for therapeutic outcome predictions in MDD. Therefore, most publications identified had to be excluded. Results of models with low performance may have been published less likely. Also, several of the evaluated publications did not provide data on performance in terms of at least AUC or accuracy. Therefore, publication and reporting bias cannot be ruled out in the present systematic evaluation.

In conclusion, the additional integration of different data categories or additional data of such categories in the course of treatment mainly improved the performance of machine learning models in predicting treatment outcomes in MDD. While this approach enhances accuracy and/or AUC, careful consideration of trade-offs between sensitivity and specificity remains crucial. Additionally, longitudinal data collection, as well as feature selection and the selection of appropriate ML-techniques tailored to the specific data types and intended use, could be involved in maximizing predictive power and improving personalized treatment outcomes for individuals with MDD. While technical awareness is evident, consistent reporting and open science practices remain limited. We recommend that future studies systematically report on missingness patterns and employ resampling or cost-sensitive learning when imbalance is present. In the case of unbalanced data distributions, AUC-PR should be reported in addition to or instead of AUC-ROC to provide a more meaningful assessment of model performance. Making trained models and pipelines publicly available will foster reproducibility. Main issues for a translational process of ML-based decision support into clinical practice remains a lack of generalizability of prediction models for clinical application in MDD and an insufficient prove of clinical applicability and utility for real-world settings.

## Data Availability

The original contributions presented in the study are included in the article/**Supplementary Material**. Further inquiries can be directed to the corresponding author.
