# Supplementary material for: Machine learning approaches in the therapeutic outcome prediction in major depressive disorder: a systematic review
Source: Front Psychiatry. 2025 Aug 13;16:1588963. doi: 10.3389/fpsyt.2025.1588963 (PMC12381684; doi:10.3389/fpsyt.2025.1588963)
Supplement: Supplementary file 1 [file Supplementaryfile1.pdf]

SUPPLEMENTARY MATERIAL 1
Machine learning methods and definitions

Table 1: Machine learning methods used in the literature selection of this review and their definitions.

Machine Learning Method	Details
**RF (Random Forest)**	A machine learning algorithm that constructs multiple decision trees during training and outputs the mode of the classes for classification or mean prediction for regression as the final output (1).
**GBDTs (Gradient Boosting Decision Trees)**	An ensemble technique that builds models sequentially, where each new model corrects the errors of the previous ones, using decision trees as the base learner (2, 3).
**CART (Classification and Regression Trees)**	A decision tree algorithm that splits the data into subsets based on feature values, creating a model for predicting either a category (classification) or a continuous outcome (regression) (4).
**GBM (Gradient Boosting Machine)**	A generalization of gradient boosting methods that optimizes model predictions by combining weak learners, typically decision trees, using a stage-wise approach (2, 3).
**XGBoost (Extreme Gradient Boosting)**	An optimized and scalable implementation of gradient boosting that includes regularization techniques to prevent overfitting and improve performance (5).
**BART (Bayesian Additive Regression Trees)**	A non-parametric Bayesian model that combines multiple regression trees, where the trees and their combination are determined by a probabilistic model, making it robust to overfitting and capable of providing uncertainty estimates (6).

Machine Learning Method	Details
**AdaBoost (Adaptive Boosting)**	A boosting technique that combines multiple weak classifiers to create a strong classifier, focusing on misclassified instances by adjusting their weights during training to improve accuracy (7).
**LASSO (Least Absolute Shrinkage and Selection Operator)**	A regression technique that performs both variable selection and regularization to enhance prediction accuracy and interpretability. It penalizes the absolute size of the coefficients, shrinking some to zero, effectively selecting a simpler model (8).
**LR (Logistic Regression)**	A statistical model used for binary classification tasks. It models the probability that a given input belongs to a particular class using a logistic function, which is a type of sigmoid function (9).
**PLR (Penalized Logistic Regression)**	A variant of logistic regression that includes a penalty (or regularization) term in the loss function, such as LASSO or Ridge, to prevent overfitting and improve model generalization (10, 11).
**GLM (Generalized Linear Model)**	A flexible generalization of ordinary linear regression that allows for the response variable to have a distribution other than a normal distribution. It includes models like logistic regression and Poisson regression (12).
**Elastic Net**	A regularized regression method that linearly combines the penalties of both LASSO (L1) and Ridge (L2). It is useful when there are multiple correlated predictors and helps in balancing between LASSO's feature selection and Ridge's regularization (13).
**SVM (Support Vector Machine)**	A supervised learning algorithm that finds the optimal hyperplane that maximizes the margin between different classes in a dataset. It's widely used for classification and regression tasks (14).
**kNN (k-Nearest Neighbors)**	A simple, instance-based learning algorithm that classifies a data point by looking at the 'k' closest data points in the feature space and taking a majority vote from the labels of those neighbors (15).

Machine Learning Method	Details
**Neural Networks (NN)**	A computational model inspired by the human brain's network of neurons. It consists of layers of nodes (neurons) that process input data through weighted connections, learning complex patterns for tasks like classification and regression (16, 17).
**Bayesian Methods**	A set of statistical methods that apply Bayes' theorem to update the probability estimates of hypotheses as more evidence or data becomes available (18).
**GNB (Gaussian Naive Bayes)**	A variant of the Naive Bayes classifier that assumes the features follow a Gaussian distribution. It's used for classification tasks, especially when the features are continuous (19).
**Naive Bayes**	A probabilistic classifier based on Bayes' theorem, assuming that the features are independent given the class label. It's simple and effective for many classification tasks (20).
**ENRR (Elastic Net Regularized Regression)**	A regularization technique that combines both LASSO (L1) and Ridge (L2) penalties in linear regression models to improve generalization and handle multicollinearity among features (13).

Table 2: Overview and definitions of performance metrics used in the identified literature for this review:

Performance metric	Details
**Accuracy**	Accuracy is the proportion of true results (both true positives and true negatives) among the total number of cases examined. Accuracy provides a general measure of how often the model is correct, but it can be misleading in cases of class imbalance (21).
**Area Under the Curve (AUC)**	Area Under the Curve (AUC) is valuable because it is invariant to the classification threshold and provides a single measure of overall performance (22).
**Specificity**	Specificity is the proportion of actual negatives that are correctly identified by the model (21).
**Sensitivity**	Sensitivity, also known as recall, is the proportion of actual positives that are correctly identified by the model (21).
**Coefficient of Determination (R^2)**	The R^2,is a statistical measure that represents the proportion of the variance in the dependent variable that is predictable from the independent variables (23).
**F1 score**	The F1 score is a crucial metric for evaluating the performance of classification models, especially when dealing with imbalanced data. It considers both false positives and false negatives, providing a more comprehensive view of model effectiveness than accuracy alone. This makes it particularly valuable in fields like fraud prevention, where accurately identifying the minority class is essential (24).

References:

1. Breiman L. Random forests. Machine learning. 2001;45:5-32.
2. Friedman JH. Greedy function approximation: a gradient boosting machine. Annals of statistics. 2001:1189-232.
3. Friedman JH. Stochastic gradient boosting. Computational statistics & data analysis. 2002;38(4):367-78.

4. Breiman L. Classification and regression trees: Routledge; 2017.
5. Chen T, Guestrin C, editors. Xgboost: A scalable tree boosting system. Proceedings of the 22nd acm sigkdd international conference on knowledge discovery and data mining; 2016.
6. Chipman HA, George EI, McCulloch RE. BART: Bayesian additive regression trees. 2010.
7. Freund Y, Schapire RE. A decision-theoretic generalization of on-line learning and an application to boosting. Journal of computer and system sciences. 1997;55(1):119-39.
8. Tibshirani R. Regression shrinkage and selection via the lasso. Journal of the Royal Statistical Society Series B: Statistical Methodology. 1996;58(1):267-88.
9. Cox DR. The regression analysis of binary sequences. Journal of the Royal Statistical Society Series B: Statistical Methodology. 1958;20(2):215-32.
10. Park MY, Hastie T. L 1-regularization path algorithm for generalized linear models. Journal of the Royal Statistical Society Series B: Statistical Methodology. 2007;69(4):659-77.
11. Goeman JJ. L1 penalized estimation in the Cox proportional hazards model. Biometrical journal Biometrische Zeitschrift. 2010;52(1):70-84.
12. Nelder JA, Wedderburn RW. Generalized linear models. Journal of the Royal Statistical Society Series A: Statistics in Society. 1972;135(3):370-84.
13. Zou H, Hastie T. Regularization and variable selection via the elastic net. Journal of the Royal Statistical Society Series B: Statistical Methodology. 2005;67(2):301-20.
14. Cortes C. Support-Vector Networks. Machine Learning. 1995.
15. Cover T, Hart P. Nearest neighbor pattern classification. IEEE transactions on information theory. 1967;13(1):21-7.
16. McCulloch WS, Pitts W. A logical calculus of the ideas immanent in nervous activity. The bulletin of mathematical biophysics. 1943;5:115-33.
17. Rumelhart DE, Hinton GE, Williams RJ. Learning representations by back-propagating errors. nature. 1986;323(6088):533-6.
18. Bayes T. LII. An essay towards solving a problem in the doctrine of chances. By the late Rev. Mr. Bayes, FRS communicated by Mr. Price, in a letter to John Canton, AMFR S. Philosophical transactions of the Royal Society of London. 1763(53):370-418.
19. de Ridder D, De Ridder J, Reinders MJ. Pattern recognition in bioinformatics. Briefings in bioinformatics. 2013;14(5):633-47.
20. McCallum A, Nigam K, editors. A comparison of event models for naive bayes text classification. AAAI-98 workshop on learning for text categorization; 1998: Madison, WI.
21. Baratloo A, Hosseini M, Negida A, El Ashal G. Part 1: Simple Definition and Calculation of Accuracy, Sensitivity and Specificity. Emergency (Tehran, Iran). 2015;3(2):48-9.
22. Hajian-Tilaki K. Receiver Operating Characteristic (ROC) Curve Analysis for Medical Diagnostic Test Evaluation. Caspian journal of internal medicine. 2013;4(2):627-35.
23. Hernandez H. Replacing the R^2 Coefficient in Model Analysis.
24. Sokolova M, Lapalme G. A systematic analysis of performance measures for classification tasks. Information Processing & Management. 2009;45(4):427-37.
