# Supplementary material for: Machine learning approaches in the therapeutic outcome prediction in major depressive disorder: a systematic review
Source: Front Psychiatry. 2025 Aug 13;16:1588963. doi: 10.3389/fpsyt.2025.1588963 (PMC12381684; doi:10.3389/fpsyt.2025.1588963)
Supplement: Supplementary file 2 [file Supplementaryfile2.pdf]

SUPPLEMENTARY MATERIAL 2

Table 1: WHO Key ethical principles for the use of AI for health and derived aspects for evaluation of clinical applicability of currently applied machine learning methods or models as diagnostic devices for outcome prediction in MDD treatment (1).

WHO principle	Derived social and ethical aspects
**Protection of autonomy**	1. **Human control:** oversight of medical decisions and health-care systems ensured<br>2. **Data protection:** Privacy and confidentiality of patient data and patients' control over their data ensured<br>3. **Human rights:** Accordance to the human rights ensured<br>4. **Informed decision:** Informed and valid consent ensured
**Promotion of human well-being, human safety and the public interest**	1. **Human well-being:** Safeguards in place to ensure that AI technologies don't harm people (continuous measurement and monitoring of the performance and to assess the impact on individuals or patient groups)<br>2. **Human safety:** Regulatory requirements for safety, accuracy and efficacy in place. Measures in place to ensure quality control and quality improvement<br>3. **Public interest:** Safeguards public interest (public health, patient need)
**Ensuring transparency, explainability and intelligibility**	1. **Intelligibility/explainability:** Ensured that AI is intelligible or understandable to developers, users and regulators<br>2. **Transparency:** Ensured that transparency includes accurate information about the assumptions of the technology and its limitations, the properties of the data, the operating protocols, and the development of the algorithmic model.
**Fostering responsibility and accountability**	1. **Responsibility:** Human warranty that regulatory principles are applied<br>2. **Accountability:** Redress including sanctions where necessary, rehabilitation, compensation, a guarantee of non-repetition and restitution
**Ensuring inclusiveness and equity**	1. **Equity:** Widest possible access and appropriate, equitable use ensured, irrespective of ability, income, age, gender or other characteristics<br>2. **Inclusiveness:** Development, monitoring and deployment of AI by employees from diverse backgrounds, cultures and disciplines. Design and evaluation with the active participation of those who will be affected by the system or are required to use it<br>3. **Diversity:** Ensuring that AI data is accurate, complete and diverse, and especially training data, do not include sampling bias<br>4. **Protection from stigmatization or discrimination**
**Promotion of artificial intelligence that is responsive and sustainable**	1. **Responsive AI:** Continuous, systematical and transparent examination of an AI technology by designers, developers and users ensured to determine an adequate and appropriate response according to communicated expectations and requirements for the intended use.<br>2. **Sustainability:** Introduction of AI technologies only if integration and sustainability in the health-care system is ensured AI approaches should be consistent with wider efforts to promote environmental and workplace sustainability and health systems<br>3. **Efficiency:** Design ensuring energy efficiency and minimization of the technology's ecological footprints

Table 2: Relevant ethical and social aspects for the use of AI in health care that are addressed by EU regulations and therefore need to be considered for an implementation of AI models in clinical settings in the EU.

**WHO (1)**	**ISO26000 (2)**	**GDPR (EU Regulation 679/2016) (3, 4)**	**MDR (EU 2017/745) (5, 6)**	**EU AI Act (7)**
Protecting human autonomy (4 aspects)	✓	✓	✓	✓
Promoting human well-being and safety and the public interest (3 aspects)	✓	✓ Aspect 1 and 3 covered, No. 2 (human safety) in terms of data safety	✓	✓
Ensuring transparency, explainability and intelligibility (2 aspects)	✓	✓	✓	✓
Fostering responsibility and accountability (2 aspects)	✓	✓	✓	✓
Ensuring inclusiveness and equity (4 aspects)	✓	✓	- Not addressed	✓
Promoting AI that is responsive and sustainable (3 aspects)	✓	- Not addressed	Aspect No. 1 (Responsive AI) only	✓

A majority of screened criteria representing WHO ethical key principles agreeing with ISO issues on social responsibility are addressed by current EU regulations that are translated into national legislation in the EU. The EU Regulations 679/2016 and EU 2017/745 do not cover criteria related to environmental sustainability and efficiency (2-3) of the principle “Promote artificial intelligence that is responsive and sustainable” as they focus on data safety or the safety and effectiveness of medical devices, respectively. However, the MDR covers one aspect of this principle in terms of responsiveness, while it does not address any of the aspects related to discrimination of the complete principle “Ensuring inclusiveness and equity”. The GDPR addresses the social/ethical aspect “Regulatory requirements for safety, accuracy and efficacy in place” (principle of well-being, safety and the public interest) only indirectly such as it refers to other guidelines in this case. However, this aspect is covered thoroughly by the MDR. A comprehensive accordance with the assessed principles was identified for the EU AI act which also covered all aspects with regard to environmental sustainability.

References:

1. WHO. Ethics and governance of artificial intelligence for health: WHO guidance. Geneva: World Health Organization; 2021.
2. ISO. ISO 26000. Social responsibility 2010 [cited 2025 17.01.2025]. Available from: https://www.iso.org/iso-26000-social-responsibility.html.
3. Regulation (EU) 2016/679 of the European Parliament and of the Council of 27 April 2016 on the protection of natural persons with regard to the processing of personal data and on the free movement of such data, and repealing Directive 95/46/EC (General Data Protection Regulation), (2016).
4. Consolidated text: Regulation (EU) 2016/679 of the European Parliament and of the Council of 27 April 2016 on the protection of natural persons with regard to the processing of personal data and on the free movement of such data, and repealing Directive 95/46/EC (General Data Protection Regulation) (Text with EEA relevance), (2016).

5. Regulation (EU) 2017/745 of the European Parliament and of the Council of 5 April 2017 on medical devices, amending Directive 2001/83/EC, Regulation (EC) No 178/2002 and Regulation (EC) No 1223/2009 and repealing Council Directives 90/385/EEC and 93/42/EEC, (2017).
6. Consolidated text: Regulation (EU) 2017/745 of the European Parliament and of the Council of 5 April 2017 on medical devices, amending Directive 2001/83/EC, Regulation (EC) No 178/2002 and Regulation (EC) No 1223/2009 and repealing Council Directives 90/385/EEC and 93/42/EEC (Text with EEA relevance), (2025).
7. Regulation (EU) 2024/1689 of the European Parliament and of the Council of 13 June 2024 laying down harmonised rules on artificial intelligence and amending Regulations (EC) No 300/2008, (EU) No 167/2013, (EU) No 168/2013, (EU) 2018/858, (EU) 2018/1139 and (EU) 2019/2144 and Directives 2014/90/EU, (EU) 2016/797 and (EU) 2020/1828 (Artificial Intelligence Act) (Text with EEA relevance), (2024).

Table 3: Literature identification from Pubmed and Google Scholar on the basis of filters, inclusion and exclusion criteria.

	Identified literature (2016-2023+2024)	Exclusion (2016-2023+2024)	Pubmed (2016-2023+2024)	Google Scholar (2016-2023+2024)
Initial number	21484 + 7114	-	84+34	21400+7080
Filter settings & duplicate removal	175+54	21.309+7060	34+3	141+51
Published in peer reviewed journal, original paper, predictions in MDD treatment, clearly listed ML method	25+1	150+53	20+1	5+0
Treatment outcome prediction	24+1	1+0	19+1	5+0
