# Supplementary material for: Machine learning approaches in the therapeutic outcome prediction in major depressive disorder: a systematic review
Source: Front Psychiatry. 2025 Aug 13;16:1588963. doi: 10.3389/fpsyt.2025.1588963 (PMC12381684; doi:10.3389/fpsyt.2025.1588963)
Supplement: Supplementary file 3 [file Supplementaryfile3.pdf]

Table 1: Performance metrics of internally validated models (approaches with largest predictor sets) using clinical-sociodemographic data and providing at least data on AUC or accuracy.

Publication	Internal validation	Pharmacological intervention	Predicted outcome	ML model	AUC	Accuracy	Sensitivity	Specificity
**Clinical-sociodemographic data**								
Iniesta et al. (2016)	10-fold cross- validation, cross-drug analysis *(largest predictor set with random patient allocation considered)*	Antidepressant (Escitalopram)	Remission	ENRR	Escitalopram: 0.75	-	-	-
		Antidepressant (Nortriptyline)	Remission	ENRR	Nortriptyline: 0.70	-	-	-
		Antidepressant (Nortriptyline+ Escitalopram)	Remission	ENRR	Escitalopram+ Nortriptyline: 0.74	-	-	-
Nie et al. (2018)	10-fold cross-validation *(full set of features considered)*	Antidepressant (Citalopram)	Treatment Resistant Depression	RF	0.78	70%	69%	71%
				GBDT	0.78	70%	69%	71%
				XGBoost	0.76	67%	72%	64%
				l2 PLR	0.69	63%	65%	62%
Sheu et al. (2023) (46)	Hold-out cross-validation *(likelihood score and inclusion of deep-learning imputed labels not considered)*	Antidepressant (SSRI, SNRI, Bupropion, and Mirtazapine)	Response	Regularized GLM	0.73	70%	83%	57%
				RF	0.73	70%	68%	72%
				GBM	0.73	69%	82%	56%
				Feed-forward DNN	0.70	67%	51%	82%
Sajjadian et al. (2023)	Nested cross- validation *(largest predictor set,,* baseline predictors, *no feature selection)*	Antidepressant (Escitalopram)	Response	Naïve Bayes	-	55%	65%	45%
				SVM	-	56%	39%	73%
Poirot et al. (2024)	Nested cross- validation, randomized K-fold cross-validation, *(largest predictor set without selection)*	Antidepressant (Sertraline)	Pretreatment Remission	XGBoost	0.48	47%	48%	45%
			Pretreatment Response	XGBoost	0.53	53%	54%	53%
			Early-Treatment Remission	XGBoost	0.62	59%	61%	58%
			Early-Treatment Response	XGBoost	0.58	55%	56%	56%

ENRR: Elastic Net Regularized Regression; RF: Random Forest; GBDT: Gradient Boosted Decision Trees; XGBoost: Extreme Gradient Boosting; PLR: Penalized Logistic Regression; GLM: General Linear Models; GBM: Gradient Boosting Machine; DNN: Deep Neural Network; SVM: Support Vector Machine

Table 2: Comparison of ML-approaches using clinical-sociodemographic and molecular biomarker data (metabolomics) and the metabolomics model with additional molecular biomarker data represented by 6 SNPs (multi-omics) in outcome predictions of combination antidepressant therapy (Joyce. et al (2021))

	Patient Set	M 1 (Metabolomics)		M 2 (Multi-omics)	
ML method performance		XGBoost AUC	Penalized regression AUC	XGBoost AUC	Penalized regression AUC
Internal validation (repeated cross-validation)	**Training Set 1:** PGRN-AMPS Escitalopram, PGRN-AMPS Citalopram, and CO-MED Escitalopram+placebo patients	0.69	0.69	0.68	0.72
Internal validation (repeated cross-validation)	**Training Set 2:** PGRN-AMPS Escitalopram, PGRN-AMPS Citalopram patients	0.68	0.68	0.72	0.72
Internal-external validation **Training Set 1**	**Testing-Set:** CO-MED Venlafaxine+Mirtazapine, Escitalopram+Bupropion patients	0.76	0.85	0.83	0.86
External validation (Cross-trial replication experiment) **Training Set 2**	**Testing-Set:** CO-MED Venlafaxine+Mirtazapine, Escitalopram+Bupropion patients	0.75	0.84	0.74	0.86

PGRN-AMPS: Pharmacogenomics Research Network Antidepressant Medication Pharmacogenomic Study, CO-MED: Combined Medications to Enhance Outcomes of Antidepressant Therapy, Model 1 (M1): Clinical data (depression related) + sociodemographic data + molecular biomarker data (metabolomics), M2: M1+ molecular biomarker data (multi-omics: metabolomics +6 functionally validated single-nucleotide polymorphisms (SNPs))

Table 3: Outcome prediction performance of applied models based on single and combined data categories. F1 scores for model 1 reflect the range of performance on the basis of eLORETA and surface-level EEG data. The mean AUC for Random Forest, calculated across all EEG bands, was 0.721 for eLORETA and 0.722 for surface-level EEG data in distinguishing antidepressant responders from non-responders (Jaworska et al. (2019)).

ML Methods	Model 1 (EEG Data (band power))		Model 2 (Clinical-demographic Data)		Model 3 (EEG + Clinical-demographic data)	
	AUC	F1 Score	AUC	F1 Score	AUC	F1 Score
RF	0.62-0.80	0.674-0.803	0.74	0.737	0.901	0.901
SVM		0.507-0.768		0.62		0.716
AdaBoost		0.576-0.775		0.715		0.838
CART		0.560-0.757		0.652		0.791
MLP		0.533-0.771		0.544		0.687
GNB		0.497-0.756		0.534		0.775

Machine Learning methods (ML): RF: Random Forest, SVM: Support Vector Machine, AdaBoost: Adaptive Boosting, CART: Classification and Regression Tree, MLP: Multi-Layer Perceptron, GNB: Gaussian Naive Bayes.

Table 4: Effect of the combination of clinical-sociodemographic and molecular biomarker data on prediction performance in MDD (Chen. B et al (2023))

ML Methods	Model 1		Model 2		Model 3	
	AUC	Accuracy	AUC	Accuracy	AUC	Accuracy
RF	0.581	57.7%	0.59	62.8%	0.611	62.5%
SVM	0.51	61.7%	0.516	61.5%	0.508	61.3%
LogiTBoost	0.569	62.4%	0.571	59.9%	0.57	59.8%
Rpart	0.508	56.3%	0.53	56.9%	0.53	56.6%
Logistic Regression	0.551	58.9%	0.524	54.4%	0.504	55.4%

Mode l: Clinical-sociodemographic data (without RFE); Model2: Molecular biomarker data (without RFE); Model 3: Molecular biomarker data + Clinical-sociodemographic data (without RFE). RF: Random Forest, SVM: Support Vector Machine, LogiTBoost: Logistic Boosting, Rpart: Recursive Partitioning and Regression Trees, Logistic Regression: Logistic Regression
