# Supplementary material for: Machine learning approaches in the therapeutic outcome prediction in major depressive disorder: a systematic review
Source: Front Psychiatry. 2025 Aug 13;16:1588963. doi: 10.3389/fpsyt.2025.1588963 (PMC12381684; doi:10.3389/fpsyt.2025.1588963)
Supplement: Supplementary file 4 [file Supplementaryfile4.pdf]

Table 1: Effect of the integration of additional data categories on prediction performance (with feature selection) based on a 134 baseline variable set preselected according to relevant published evidence of predictive value (Sajjandian et al. (2023))

	ML Methods (feature selection)	Model1				Model 2				Model 3				Model 4			
		AUC	Balanced Accuracy	Sensitivity	Specificity	AUC	Balanced Accuracy	Sensitivity	Specificity	AUC	Balanced Accuracy	Sensitivity	Specificity	AUC	Balanced Accuracy	Sensitivity	Specificity
Week0	RF Embedded + CAT score		52%	41%	63%		53%	47%	59%		56%	43%	69%		58%	55%	62%
Week0	SVM (CAT score)		56%	50%	61%		61%	48%	74%		54%	42%	66%		60%	54%	66%
Week0	GBM (Embedded + CAT score)		51%	43%	58%		52%	45%	59%		52%	44%	61%		56%	56%	57%
Week0	Naive Bayes (CAT score)	0.58	58%	56%	60%		52%	74%	30%		54%	49%	58%		55%	87%	23%
Week0	Elastic Net (Embedded + CAT score)		54%	43%	66%		55%	43%	67%		51%	42%	60%		59%	57%	62%
Week0+2	RF Embedded + CAT score		64%	55%	73%		54%	45%	63%		52%	43%	61%		63%	54%	72%
Week0+2	SVM (CAT score)		63%	52%	75%		56%	44%	69%		50%	40%	60%		63%	54%	73%
Week0+2	GBM Embedded + CAT score		57%	51%	63%		54%	43%	65%		49%	45%	53%		57%	49%	65%
Week0+2	Naive Bayes (CAT score)		65%	65%	65%		55%	76%	33%		54%	51%	58%		58%	61%	55%
Week0+2	Elastic Net Embedded + CAT score		62%	54%	71%		54%	46%	63%		49%	41%	56%		64%	56%	73%

CAT score: correlation-adjusted t-score; RF: Random Forest; SVM: Support Vector Machine; GBM: Gradient Boosting Machine, Model 1: Clinical -sociodemographic data, Model 2: Molecular biomarker data Model 3:MRI (multimodal and functional) data, Model 4: combination of all 3 data categories. Further combinations showing an increase of prediction accuracy with an increase of the number of data categories, are provided by Sajjandian et al. (2023)
